# Supplementary material for: Modeling glioblastoma heterogeneity as a dynamic network of cell states
Source: Mol Syst Biol. 2021 Sep 16;17(9):e10105. doi: 10.15252/msb.202010105 (PMC8444284; doi:10.15252/msb.202010105)
Supplement: Supplementary file 6 — Source Data for Figure 5 [file MSB-17-e10105-s004.zip › Figure5A_sourcedata/GSEA_3017/nosigdb_stateD.GseaPreranked.1622795010397/DOROSZKO_U3013MG_CLUSTER_2.html]

Details for gene set DOROSZKO\_U3013MG\_CLUSTER\_2[GSEA]

|  || Dataset | state23017 |
| Phenotype | NoPhenotypeAvailable |
| Upregulated in class | na\_neg |
| GeneSet | DOROSZKO\_U3013MG\_CLUSTER\_2 |
| Enrichment Score (ES) | -0.55749744 |
| Normalized Enrichment Score (NES) | -3.1988902 |
| Nominal p-value | 0.0 |
| FDR q-value | 0.0 |
| FWER p-Value | 0.0 |
Table: GSEA Results Summary

  

Fig 1: Enrichment plot: DOROSZKO\_U3013MG\_CLUSTER\_2      
 Profile of the Running ES Score & Positions of GeneSet Members on the Rank Ordered List

  

| PROBE | GENE SYMBOL | GENE\_TITLE | RANK IN GENE LIST | RANK METRIC SCORE | RUNNING ES | CORE ENRICHMENT || 1 | PTTG1 |  |  | 14 | 0.490 | -0.0054 | No |
| 2 | HDGF |  |  | 58 | 0.338 | -0.0374 | No |
| 3 | HMGB3 |  |  | 80 | 0.311 | -0.0511 | No |
| 4 | TROAP |  |  | 109 | 0.283 | -0.0710 | No |
| 5 | LSM5 |  |  | 123 | 0.274 | -0.0784 | No |
| 6 | BIRC5 |  |  | 130 | 0.271 | -0.0799 | No |
| 7 | MKI67 |  |  | 206 | -0.254 | -0.1402 | No |
| 8 | NSD2 |  |  | 259 | -0.260 | -0.1808 | No |
| 9 | TRIP13 |  |  | 294 | -0.263 | -0.2062 | No |
| 10 | HMMR |  |  | 319 | -0.266 | -0.2230 | No |
| 11 | UACA |  |  | 323 | -0.266 | -0.2220 | No |
| 12 | EXOSC8 |  |  | 347 | -0.268 | -0.2380 | No |
| 13 | PRR11 |  |  | 361 | -0.269 | -0.2454 | No |
| 14 | MASTL |  |  | 414 | -0.275 | -0.2859 | No |
| 15 | NEURL1B |  |  | 454 | -0.280 | -0.3153 | No |
| 16 | TEDC1 |  |  | 462 | -0.281 | -0.3175 | No |
| 17 | CDC25B |  |  | 472 | -0.282 | -0.3214 | No |
| 18 | APOLD1 |  |  | 476 | -0.282 | -0.3202 | No |
| 19 | FOXM1 |  |  | 478 | -0.282 | -0.3173 | No |
| 20 | ECT2 |  |  | 481 | -0.283 | -0.3152 | No |
| 21 | PSRC1 |  |  | 574 | -0.295 | -0.3893 | No |
| 22 | BTG3 |  |  | 581 | -0.296 | -0.3905 | No |
| 23 | SPAG5 |  |  | 605 | -0.301 | -0.4060 | No |
| 24 | PRC1 |  |  | 645 | -0.310 | -0.4350 | No |
| 25 | CDK5RAP2 |  |  | 651 | -0.311 | -0.4351 | No |
| 26 | RAD21 |  |  | 674 | -0.314 | -0.4496 | No |
| 27 | TMPO |  |  | 688 | -0.317 | -0.4564 | No |
| 28 | KNSTRN |  |  | 690 | -0.317 | -0.4530 | No |
| 29 | KIF4A |  |  | 706 | -0.320 | -0.4615 | No |
| 30 | SPC24 |  |  | 747 | -0.330 | -0.4911 | No |
| 31 | SOGA1 |  |  | 757 | -0.333 | -0.4943 | No |
| 32 | CDC20 |  |  | 793 | -0.341 | -0.5194 | No |
| 33 | FANCI |  |  | 801 | -0.345 | -0.5208 | No |
| 34 | H2AFX |  |  | 810 | -0.347 | -0.5230 | No |
| 35 | NCAPD2 |  |  | 811 | -0.347 | -0.5184 | No |
| 36 | CENPM |  |  | 818 | -0.349 | -0.5188 | No |
| 37 | MAD2L1 |  |  | 819 | -0.350 | -0.5142 | No |
| 38 | PHIP |  |  | 823 | -0.350 | -0.5121 | No |
| 39 | BUB3 |  |  | 826 | -0.351 | -0.5091 | No |
| 40 | CBX5 |  |  | 842 | -0.356 | -0.5171 | No |
| 41 | SHCBP1 |  |  | 844 | -0.356 | -0.5133 | No |
| 42 | RFC3 |  |  | 852 | -0.358 | -0.5144 | No |
| 43 | NEK2 |  |  | 861 | -0.359 | -0.5165 | No |
| 44 | CENPQ |  |  | 884 | -0.367 | -0.5303 | No |
| 45 | CKAP2 |  |  | 900 | -0.375 | -0.5380 | No |
| 46 | TK1 |  |  | 902 | -0.375 | -0.5339 | No |
| 47 | SAP30 |  |  | 905 | -0.376 | -0.5306 | No |
| 48 | TUBB4B |  |  | 916 | -0.380 | -0.5340 | No |
| 49 | USP1 |  |  | 930 | -0.386 | -0.5399 | No |
| 50 | CDKN2D |  |  | 942 | -0.390 | -0.5441 | No |
| 51 | DIAPH3 |  |  | 943 | -0.390 | -0.5389 | No |
| 52 | SKA3 |  |  | 965 | -0.399 | -0.5514 | No |
| 53 | XPO1 |  |  | 967 | -0.400 | -0.5470 | No |
| 54 | HIRIP3 |  |  | 970 | -0.402 | -0.5433 | No |
| 55 | RTKN2 |  |  | 973 | -0.403 | -0.5397 | No |
| 56 | SPDL1 |  |  | 995 | -0.412 | -0.5520 | Yes |
| 57 | FAM83D |  |  | 998 | -0.413 | -0.5483 | Yes |
| 58 | KIF11 |  |  | 1003 | -0.416 | -0.5461 | Yes |
| 59 | MNS1 |  |  | 1009 | -0.419 | -0.5448 | Yes |
| 60 | CCP110 |  |  | 1010 | -0.420 | -0.5392 | Yes |
| 61 | CCDC34 |  |  | 1013 | -0.421 | -0.5354 | Yes |
| 62 | SAC3D1 |  |  | 1025 | -0.429 | -0.5390 | Yes |
| 63 | NUCB2 |  |  | 1026 | -0.430 | -0.5333 | Yes |
| 64 | TPX2 |  |  | 1034 | -0.437 | -0.5334 | Yes |
| 65 | KIF22 |  |  | 1051 | -0.446 | -0.5411 | Yes |
| 66 | SMC2 |  |  | 1062 | -0.452 | -0.5436 | Yes |
| 67 | CDKN2C |  |  | 1067 | -0.454 | -0.5409 | Yes |
| 68 | EZH2 |  |  | 1070 | -0.455 | -0.5366 | Yes |
| 69 | DEPDC1 |  |  | 1074 | -0.457 | -0.5331 | Yes |
| 70 | PSMC3IP |  |  | 1075 | -0.457 | -0.5270 | Yes |
| 71 | PLK1 |  |  | 1082 | -0.460 | -0.5260 | Yes |
| 72 | MND1 |  |  | 1083 | -0.461 | -0.5199 | Yes |
| 73 | ATAD5 |  |  | 1091 | -0.465 | -0.5196 | Yes |
| 74 | ITGB3BP |  |  | 1096 | -0.467 | -0.5168 | Yes |
| 75 | ZWINT |  |  | 1099 | -0.467 | -0.5123 | Yes |
| 76 | BUB1B |  |  | 1111 | -0.477 | -0.5153 | Yes |
| 77 | RACGAP1 |  |  | 1112 | -0.479 | -0.5090 | Yes |
| 78 | TTK |  |  | 1119 | -0.487 | -0.5076 | Yes |
| 79 | NCAPD3 |  |  | 1130 | -0.496 | -0.5095 | Yes |
| 80 | ASF1B |  |  | 1133 | -0.498 | -0.5046 | Yes |
| 81 | CKAP2L |  |  | 1147 | -0.508 | -0.5089 | Yes |
| 82 | MXD3 |  |  | 1153 | -0.514 | -0.5063 | Yes |
| 83 | NUSAP1 |  |  | 1154 | -0.515 | -0.4995 | Yes |
| 84 | GMNN |  |  | 1155 | -0.516 | -0.4926 | Yes |
| 85 | DBF4 |  |  | 1157 | -0.520 | -0.4866 | Yes |
| 86 | MIS18BP1 |  |  | 1162 | -0.524 | -0.4830 | Yes |
| 87 | CIP2A |  |  | 1165 | -0.533 | -0.4776 | Yes |
| 88 | RRM1 |  |  | 1166 | -0.534 | -0.4706 | Yes |
| 89 | KNL1 |  |  | 1167 | -0.534 | -0.4635 | Yes |
| 90 | KIF14 |  |  | 1168 | -0.535 | -0.4564 | Yes |
| 91 | BRCA1 |  |  | 1171 | -0.541 | -0.4509 | Yes |
| 92 | SGO2 |  |  | 1176 | -0.546 | -0.4471 | Yes |
| 93 | ORC6 |  |  | 1179 | -0.551 | -0.4414 | Yes |
| 94 | CENPU |  |  | 1182 | -0.555 | -0.4358 | Yes |
| 95 | KIF2C |  |  | 1183 | -0.558 | -0.4284 | Yes |
| 96 | CDCA3 |  |  | 1187 | -0.563 | -0.4234 | Yes |
| 97 | ASPM |  |  | 1189 | -0.567 | -0.4168 | Yes |
| 98 | FEN1 |  |  | 1191 | -0.569 | -0.4101 | Yes |
| 99 | NUF2 |  |  | 1194 | -0.574 | -0.4041 | Yes |
| 100 | DHFR |  |  | 1197 | -0.576 | -0.3982 | Yes |
| 101 | CKS1B |  |  | 1198 | -0.577 | -0.3905 | Yes |
| 102 | GTSE1 |  |  | 1210 | -0.602 | -0.3919 | Yes |
| 103 | CLSPN |  |  | 1212 | -0.605 | -0.3847 | Yes |
| 104 | KIF15 |  |  | 1213 | -0.610 | -0.3766 | Yes |
| 105 | CENPK |  |  | 1220 | -0.619 | -0.3735 | Yes |
| 106 | C21orf58 |  |  | 1222 | -0.626 | -0.3660 | Yes |
| 107 | SMC4 |  |  | 1224 | -0.638 | -0.3584 | Yes |
| 108 | MYBL2 |  |  | 1225 | -0.639 | -0.3500 | Yes |
| 109 | CKS2 |  |  | 1226 | -0.641 | -0.3414 | Yes |
| 110 | NCAPH |  |  | 1228 | -0.643 | -0.3338 | Yes |
| 111 | ANLN |  |  | 1229 | -0.646 | -0.3252 | Yes |
| 112 | CDCA8 |  |  | 1239 | -0.669 | -0.3239 | Yes |
| 113 | CENPA |  |  | 1243 | -0.679 | -0.3175 | Yes |
| 114 | CDCA5 |  |  | 1244 | -0.685 | -0.3084 | Yes |
| 115 | SGO1 |  |  | 1246 | -0.691 | -0.3001 | Yes |
| 116 | AURKB |  |  | 1250 | -0.700 | -0.2933 | Yes |
| 117 | MELK |  |  | 1251 | -0.701 | -0.2840 | Yes |
| 118 | CCNA2 |  |  | 1255 | -0.716 | -0.2771 | Yes |
| 119 | UBE2T |  |  | 1256 | -0.719 | -0.2675 | Yes |
| 120 | CDCA2 |  |  | 1258 | -0.727 | -0.2587 | Yes |
| 121 | ARHGAP11A |  |  | 1262 | -0.735 | -0.2515 | Yes |
| 122 | HMGB2 |  |  | 1263 | -0.736 | -0.2418 | Yes |
| 123 | RAD51AP1 |  |  | 1264 | -0.741 | -0.2319 | Yes |
| 124 | KIF23 |  |  | 1265 | -0.741 | -0.2221 | Yes |
| 125 | KIFC1 |  |  | 1267 | -0.748 | -0.2130 | Yes |
| 126 | TACC3 |  |  | 1268 | -0.751 | -0.2031 | Yes |
| 127 | AURKA |  |  | 1271 | -0.763 | -0.1946 | Yes |
| 128 | TYMS |  |  | 1272 | -0.770 | -0.1844 | Yes |
| 129 | ATAD2 |  |  | 1275 | -0.775 | -0.1758 | Yes |
| 130 | NDC80 |  |  | 1278 | -0.810 | -0.1668 | Yes |
| 131 | PCLAF |  |  | 1281 | -0.828 | -0.1575 | Yes |
| 132 | ESCO2 |  |  | 1283 | -0.846 | -0.1471 | Yes |
| 133 | FAM111A |  |  | 1285 | -0.871 | -0.1364 | Yes |
| 134 | HJURP |  |  | 1290 | -0.897 | -0.1279 | Yes |
| 135 | SPC25 |  |  | 1294 | -0.932 | -0.1181 | Yes |
| 136 | RRM2 |  |  | 1295 | -0.933 | -0.1057 | Yes |
| 137 | KPNA2 |  |  | 1297 | -0.972 | -0.0937 | Yes |
| 138 | NCAPG |  |  | 1303 | -1.015 | -0.0844 | Yes |
| 139 | CDK1 |  |  | 1311 | -1.433 | -0.0714 | Yes |
| 140 | UBE2C |  |  | 1312 | -1.466 | -0.0519 | Yes |
| 141 | TOP2A |  |  | 1313 | -1.528 | -0.0316 | Yes |
| 142 | HIST1H4C |  |  | 1320 | -2.769 | 0.0000 | Yes |
Table: GSEA details [plain text format]

  

Fig 2: DOROSZKO\_U3013MG\_CLUSTER\_2: Random ES distribution      
 Gene set null distribution of ES for **DOROSZKO\_U3013MG\_CLUSTER\_2**
